# Genetic control of morphological traits useful for improving sorghum

**DOI:** 10.1270/jsbbs.22069

**Published:** 2023-01-17

**Authors:** Hideki Takanashi

**Affiliations:** 1 Graduate School of Agricultural and Life Sciences, The University of Tokyo, 1-1-1 Yayoi, Bunkyo-ku, Tokyo 113-8657, Japan

**Keywords:** sorghum, spikelet, grain, biomass, breeding, morphological traits

## Abstract

Global climate change and global warming, coupled with the growing population, have raised concerns about sustainable food supply and bioenergy demand. Sorghum [*Sorghum bicolor* (L.) Moench] ranks fifth among cereals produced worldwide; it is a C_4_ crop with a higher stress tolerance than other major cereals and has a wide range of uses, such as grains, forage, and biomass. Therefore, sorghum has attracted attention as a promising crop for achieving sustainable development goals (SDGs). In addition, sorghum is a suitable genetic model for C_4_ grasses because of its high morphological diversity and relatively small genome size compared to other C_4_ grasses. Although sorghum breeding and genetic studies have lagged compared to other crops such as rice and maize, recent advances in research have identified several genes and many quantitative trait loci (QTLs) that control important agronomic traits in sorghum. This review outlines traits and genetic information with a focus on morphogenetic aspects that may be useful in sorghum breeding for grain and biomass utilization.

## Introduction

The world population continues to grow and is expected to reach 9.7 billion by 2050. In addition, because of climate change, global temperature is on the rise, and the occurrence of droughts and floods has become more frequent and widespread (Rivero *et al.* 2022). Thus, there is a pressing need to develop solutions for sustainable food supply and bioenergy under a changing global climate. One such solution is the use of high-biomass crops that not only provide cereal foods and forage but also feedstocks for biofuel production from starch, sugar, and lignocellulosic substances. In particular, Andropogoneae members, including maize and sorghum, are considered promising species (Paterson *et al.* 2009, Rooney *et al.* 2007, van der Weijde *et al.* 2013).

Sorghum [*Sorghum bicolor* (L.) Moench] is believed to have originated in sub-Saharan Africa; the species has since diversified to thrive under tropical to temperate climates (Balota *et al.* 2008, Peng and Krieg 1992), resulting in sorghum being the fifth-highest cereal cultivated worldwide (faostat.fao.org). It is also known that sorghum is a C_4_ crop with higher stress tolerance than other major cereals (Ogbaga *et al.* 2014, Tuinstra *et al.* 1997). Furthermore, sorghum has widespread uses, including as grains for food and feed (grain/sweet sorghum), forage (forage/sweet sorghum), and biomass for bioenergy (biomass/sweet sorghum). Thus, sorghum has attracted attention as a promising crop that contributes to sustainable development goals (SDGs) (Geisen *et al.* 2021, Hao *et al.* 2018). In addition, sorghum is a suitable genetic model for C_4_ grasses because of its high morphological diversity and relatively small genome size (approximately 732 Mb) compared to those of other C_4_ grasses (McCormick *et al.* 2018, Paterson *et al.* 2009, Price *et al.* 2005).

Although sorghum can be used for various applications, the ideal traits required for each application are different; therefore, appropriate sorghum varieties must be bred corresponding to the particular use. To this end, it is essential to understand the loci and genes that control the trait to be improved. Chromosomal regions responsible for genetic variance of the traits interest can be mapped as quantitative trait loci (QTLs) and quantitative trait nucleotides using QTL analysis and genome-wide association studies (GWAS); these genetic loci contribute to crop improvement via marker-assisted selection (Collard and Mackill 2008). Furthermore, marker-trait associations have been shown to facilitate the implementation of genomic selection for quantitative traits (Liu *et al.* 2019).

Unlike rice and maize, limited information is available on loci and genes that control traits in sorghum. Nevertheless, recent advances in research have identified several genes and QTLs that control important agronomic traits in sorghum. This review outlines traits and genetic information with a focus on morphogenetic aspects that may be useful in sorghum breeding for grain and biomass utilization. In this review, sorghum gene IDs are denoted as Sobic.00XGYYYYYY based on the sorghum reference genome v3.1.1 (McCormick *et al.* 2018) in the Phytozome database (https://phytozome-next.jgi.doe.gov/info/Sbicolor_v3_1_1).

## Useful traits for grain sorghum

**Fig. 1.**
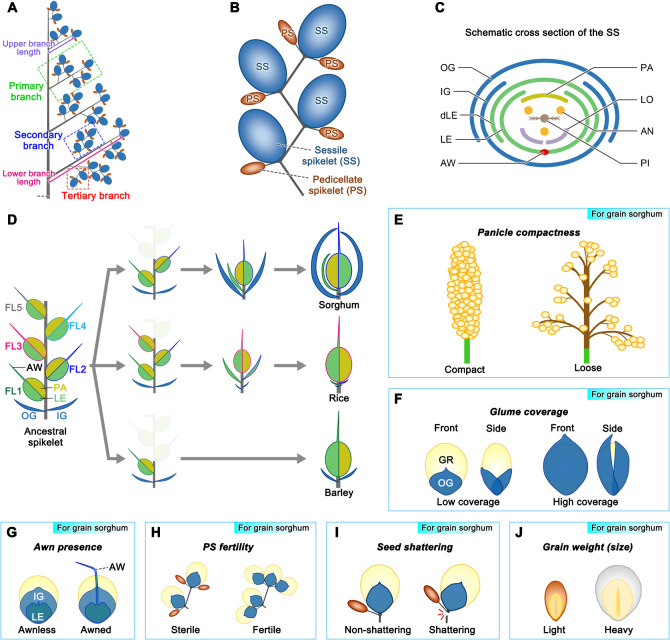
Useful traits for grain sorghum. (A) Scheme of the sorghum panicle (only the right side of the panicle is depicted). (B) An example of a secondary branch of the sorghum panicle. SS: sessile spikelet, and PS: pedicellate spikelet. (C) Schematic diagram of the internal structure of the SS in sorghum. OG: outer glume, IG: inner glume, dLE: lemma of a degenerated floret, LE: lemma with awn (AW), PA: palea, LO: lodicule, AN: anther, and PI: pistil. (D) Estimated evolutionary processes of the spikelet structures in sorghum, rice, and barley. FL: floret. Only FL2, FL3, and FL1 are considered to remain as functional florets during evolution in sorghum, rice, and barley, respectively. (E–J) Examples of useful traits for grain sorghum: panicle compactness (E), glume coverage (F), awn presence (G), PS fertility (H), seed shattering (I), and grain weight (J). GR: grain.

Sorghum grain is a staple food for over 500 million people in the semi-arid tropics of Asia and Africa. In developed countries, such as the USA and Australia, it is the main source of livestock feed and industrial use. Sorghum grains, like those of other grasses, are enfolded into floral bracts (glumes, lemmas, and paleas) that form a terminal unit called a spikelet. This section presents traits that may be useful for grain sorghum (Fig. 1), including spikelet morphology.

### Panicle-related traits

Panicle morphology is a key component of crop adaptation and yield (Cooper *et al.* 2014, Harlan and de Wet 1972); its morphology depends on the number and length of panicle branches (Brown *et al.* 2006, Fig. 1A). Cultivated sorghum is classiﬁed into ﬁve major races (bicolor, guinea, caudatum, durra, and kaﬁr) mainly based on panicle and spikelet characteristics (Harlan and de Wet 1972). A compact panicle is typical of domesticated sorghum (Brown *et al.* 2006, Fig. 1E left), whereas undomesticated species are more likely to have loose panicles (Harlan and de Wet 1972, Fig. 1E right). It is known that compact panicles are more prone to infection/infestation by grain molds (Sharma *et al.* 2010), webworms (Hobbs *et al.* 1979), head bugs, and head caterpillars (Sharma *et al.* 1994). To prevent grain molding, race guinea with loose panicles is more common in wet environments, while race durra with compact panicles is mainly cultivated in dry environments (Ayana and Bekele 1998, Harlan and de Wet 1972).

Recently, Zhou *et al.* (2019) performed a GWAS for panicle-related traits such as panicle length, panicle width, and panicle compactness (referred to as solidity) using 272 genotypes comprising a subset of the Sorghum Association Panel (SAP: Casa *et al.* 2008) and a high-resolution imaging pipeline. They detected 35 unique single-nucleotide polymorphisms (SNPs) associated with variations in panicle architecture and identified three candidate genes responsible for panicle length: *Sobic.001G106200* (homeobox domain-containing protein), *Sobic.002G247800* (SBP-box gene family member), and *Sb03g030635* (Homeobox3; *Sb03g030635* is an old gene ID, and there is no corresponding gene in the current v3.1.1 reference genome); two for panicle width: *Sobic.003G027000* (MADS-box family gene with MIKCc-type box) and *Sobic.010G267700* (receptor protein kinase CLAVATA1-like gene); three for panicle solidity: *Sobic.001G214000* (Dicer-like3), *Sb02g042400* (AP2 domain-containing protein; *Sb02g042400* is an old gene ID, and there is no corresponding gene in the current v3.1.1 reference genome), and *Sobic.006G254900* (homeobox domain-containing protein). Olatoye *et al.* (2020) also characterized the genetic architecture of four panicle morphological traits [upper branch length (UBL), lower branch length (LBL), rachis length (RL), and rachis diameter (RD); ranging from 7 to 170, 176 to 267, 111 to 465, and 3.8 to 13.5 (mm), respectively] using a nested association mapping (NAM) population derived from a cross between an elite U.S. common parent RTx430 and ten diverse founders. They showed that the proportion of within-family variation explained by all QTLs varied among traits, with 12%, 37%, 31%, and 21% of variation explained by QTLs for UBL, LBL, RL, and RD, respectively. Some of the detected QTLs were colocalized with known panicle-related gene homologs. For example, they reported *Sobic.001G219400* (rice *TAWAWA1* ortholog: for RL), *Sobic.003G052900* (maize *Ramosa2* ortholog: for UBL), and *Sobic.010G220400* (rice *Aberrant Panicle Organization1* ortholog: for LBL and RL) as candidate genes responsible for the regulation of panicle morphology. In addition to the above genes, there were many QTLs with no hits for homologs of known panicle-related genes (Olatoye *et al.* 2020), suggesting that a number of unknown genes are involved in sorghum panicle morphogenesis. Further functional studies such as ﬁne mapping, mutant analysis, and gene expression analysis are required to identify these responsible genes.

### Sessile spikelet-related traits

Sorghum has two types of spikelets: a sessile spikelet (SS), and a pedicellate spikelet (PS; Fig. 1B); among the two, only SSs are fertile, therefore, SS-related traits are discussed here. A sorghum SS seemingly produces two florets, positioned as upper and lower florets. The lower floret degenerates leaving only the lemma; eventually, only the upper floret is fertile (Fig. 1C), resulting in a “single grain per spikelet” type. Rice and barley also fall under the “single grain per spikelet” type, but the evolutionary processes leading to the establishment of their spikelet structures are largely different from those of sorghum (Wang *et al.* 2022b, Fig. 1D).

In sorghum SS, glumes are the outermost organs protecting the grain (Fig. 1D); however, the grains tend to protrude from the glume in many grain sorghum varieties. Future improvements in glume characteristics may be important as some reports have indicated that the size and hardness of the glume play a role in pest resistance (Rossetto *et al.* 1984, Sharma *et al.* 1990). Several QTLs related to the ratio of grain coverage by glumes have been identified in sorghum (Feltus *et al.* 2006, Girma *et al.* 2019, Fig. 1F) on chromosomes (chr) 1, 2, 3, and 6. Xie *et al.* (2022) has recently identified one responsible gene, *Glume Coverage 1* (*GC1*) using GWAS and map-based cloning. *GC1* (*Sobic.001g341700*) encodes an atypical G protein γ subunit and negatively regulates sorghum glume coverage by mainly affecting glume length. They found that *GC1* is an ortholog of the rice grain size-regulating gene *GS3* (Fan *et al.* 2006, Mao *et al.* 2010). Takanashi *et al.* (2021) performed QTL analysis using a sorghum recombinant inbred line (RIL) population derived from a cross between BTx623 and Japanese landrace NOG for glume (spikelet) length (ranging from 4.38 to 6.07 mm). They reported two reproducible QTLs on chr6 (*ca.* 49.0 Mb, explaining 10.2% of the phenotypic variation; hereinafter referred to as “PVE: 10.2%”) and chr9 (*ca.* 57.2 Mb, PVE: 17.0%); the candidate gene at chr9 was identified to be *Dw1*, a known gene related to plant height.

The awn, a needle-like structure that forms at the tip of the lemma in grass species, plays a role in environmental adaptation and fitness (Fig. 1G). In sorghum, varieties with awns are more resistant to bird attacks than those without awns (Mofokeng and Shargie 2016). Meanwhile, the selection of awnless crops was a key factor during crop improvements, as awnless grains facilitate grain harvest and storage. In sorghum, a dominant awn-inhibiting locus has been identified in 1921 (Vinall and Cron 1921); however, the gene responsible for awn-inhibition has long remained unidentified. Recently, the gene responsible for the dominant awn-inhibiting locus, *awn1* [also known as *DOMINANT AWN INHIBITOR* (*DAI*); hereinafter referred to as *awn1*/*DAI*] (*Sobic003G421300*), was identified (Takanashi *et al.* 2022, Zhou *et al.* 2021). *awn1*/*DAI*, which encodes the ALOG family protein, originated from the sorghum-specific gene duplication of its twin paralog on chr10. awn1/DAI downregulates the expression of three MADS-box genes, *MADS3*, *MADS6*, and *MADS7*, as well as sorghum orthologs for *DROOPING LEAF* and *LKS2* involved in awn development in rice and barley (Zhou *et al.* 2021), and inhibits awn elongation by suppressing both cell proliferation and elongation (Takanashi *et al.* 2022). Interestingly, heterologous expression of sorghum *awn1*/*DAI* under its own promoter inhibited awn elongation in the awned rice cultivar Kasalath, suggesting the existence of a common awn-inhibition mechanism in sorghum and rice (Takanashi *et al.* 2022). In addition to *awn1*/*DAI*, several other QTLs related to awn length in awned sorghum lines [ranging from 1.0 to 13.5 mm in Hart *et al.* (2001); 6.6 to 16.7 mm in Takanashi *et al.* (2022)] have been reported on chr6 (*ca.* 56.9 Mb, PVE: 6.7%), chr7 (*ca.* 59.7 Mb, PVE: 23.8%), and chr9 (*ca.* 57.7 Mb, PVE: 16.2%), and Takanashi *et al.* (2022) suggested that the candidate genes responsible for QTLs on chr7 and chr9 are the genes *Dw3* and *Dw1*, which relate to plant height.

The morphologies of other SS-related organs such as the lodicule, anther, and pistil (ovary with style and stigma), are also important for sexual reproduction in grasses. For example, the efficiency of hybrid seed production can be improved by increasing the percentage of exserted stigma, which is closely related to the balance between spikelet size and stigma length in rice (Liu *et al.* 2015). By contrast, cleistogamy, which refers to autonomous self-pollination caused by the failure of spikelets to open, is a useful genetic tool for preventing possible gene transfer in transgenic crops (Daniell 2002), which maintains the genetic purity of inbreds across generations (Saxena *et al.* 1992). The establishment of cleistogamy may be affected by the percentage of exserted stigma, anther length, and lodicule size. Although genetic studies on the morphologies of these spikelet-related organs in sorghum are limited, a QTL for anther length (ranging from 2.39 to 3.99 mm) on chr9 (*ca.* 57.2 Mb, PVE: 14.8%), two QTLs for style length (ranging from 1.03 to 2.22 mm) on chr6 (*ca.* 48.1 Mb, PVE: 16.1%) and chr7 (*ca.* 59.7 Mb, PVE: 13.1%), two QTLs for stigma length (ranging from 0.82 to 2.42 mm) on chr1 (*ca.* 80.1 Mb, PVE: 9.6%) and chr3 (*ca.* 73.1 Mb, PVE: 25.7%), and three QTLs for stigma width (ranging from 0.22 to 0.83 mm) on chr3 (*ca.* 1.0 Mb, PVE: 14.5%), chr6 (*ca.* 48.1 Mb, PVE: 9.2%), and chr9 (*ca.* 57.2 Mb, PVE: 12.0%) were recently reported using a RIL population derived from a cross between BTx623 and NOG (Takanashi *et al.* 2021). It has been suggested that the candidate genes responsible for QTLs on chr7 (style length) and chr9 (anther length and stigma width) are also *Dw3* and *Dw1* (Takanashi *et al.* 2021). This report is valuable because it is the first report on the loci that control the size of these organs in sorghum. However, since the results were obtained using a single RIL population, it is yet to be known if they can actually be used in breeding. Future improvement of these traits will require careful consideration of the results obtained using different types of populations.

### Pedicellate spikelet-related traits

In sorghum germplasm, PS has an incomplete internal floret structure and is sterile (Karper and Stephens 1936). Three *multi-seed* (*msd*) mutants that produce normal grains in both SS and PS were recently isolated from an ethyl methanesulfonate (EMS)-induced mutant population (Fig. 1H); their responsible genes were identified as *MSD1*, *MSD2*, and *MSD3* (Dampanaboina *et al.* 2019, Gladman *et al.* 2019, Jiao *et al.* 2018). *MSD1* (*Sobic.007G135700*) encodes a TCP transcription factor that activates several enzymes related to the jasmonic acid (JA) biosynthetic pathway (Jiao *et al.* 2018). *MSD2* (*Sobic.006G095600*) encodes a 13-lipoxygenase, involved in the first step of JA biosynthesis (Gladman *et al.* 2019). *MSD1* binds to the upstream region of the transcriptional start site of itself and *MSD2* to control the expression. *MSD3* (*Sobic.001G407600*) encodes a linoleic desaturase FAD7 which is required for the biosynthesis of the substrate for JA biosynthesis (Dampanaboina *et al.* 2019). Recessive alleles of *MSD1*, *MSD2*, and *MSD3* promote the development of PSs and set grains, showing that JA plays an important role in the development of PS. Burow *et al.* (2014) reported that the number of grains per panicle in the *msd1* mutant was 3.6 times higher than that in the wild-type because of the increased length of the primary branch of the panicle in addition to the grain setting of PSs. Although the 100-grain weight in the *msd1* mutant was reduced to ~50% of the wild type due in part to trade-offs, the total grain yield (g) per panicle in the mutant was reported to be 37% higher than that in the wild-type because of the significant increase in the number of grains in the mutant. These results suggest that *msd1* mutant is a useful tool for improving grain yield. Recently, it was reported that the PS contributes to grain weight by translocating its photosynthetic products to the SS (AuBuchon-Elder *et al.* 2020), suggesting that the PS is not a “useless” organ even in sorghum germplasm with sterile PSs.

### Grain yield

Grain yield (GY) is one of the most important traits for grain sorghum; numerous QTLs (over 180) pertaining to GY have been identified (Mace *et al.* 2019). Seed shattering, grain number per panicle (GNP), grain weight (thousand-grain weight: TGW; Fig. 1J), and tiller number (described in a later section) are the major factors affecting GY.

The first gene that regulates seed shattering in sorghum (Fig. 1I) was identified as *Sh1* (*Sobic.001G152901*), which encodes a YABBY transcription factor (Lin *et al.* 2012). Non-shattering sorghum accessions harbored three different mutations at the *Sh1* locus; variants at regulatory sites in the promoter and intronic regions led to a low level of expression, a 2.2-kb deletion caused a truncated transcript that lacked exons 2 and 3, and a GT-to-GG splice-site variant in intron 4 resulted in the removal of exon 4. Tang *et al.* (2013) revealed that a WRKY transcription factor, SpWRKY, conferred shattering to a wild sorghum relative, *Sorghum propinquum*, which is only 300 kb apart from *Sh1*. Recessive SbWRKY (Sobic.001G148000) is 44 amino acids shorter than the dominant SpWRKY because of the difference in the location of start codon. Sh1 and WRKY are likely involved in forming the abscission zone in the grain-pedicel junction, similar to the known causes of seed shuttering in rice; however, further analysis is needed to elucidate these details.

Boyles *et al.* (2017) performed QTL analysis for GNP using two biparental sorghum RIL populations derived from a cross between BTx642 and BTxARG-1 (ranging from 999 to 3,716) and a cross between P850029 and BTxARG-1 (ranging from 1,132 to 4,026). They detected seven QTLs for GNP on chr1 (*ca.* 0.8 Mb, PVE: 11.2%; *ca.* 1.5 Mb, PVE: 21.4%), chr3 (*ca.* 7.1 Mb, PVE: 5.5%; *ca.* 56.5 Mb, PVE: 5.6%), chr4 (*ca.* 67.4 Mb, PVE: 4.8%), chr5 (*ca.* 66.8 Mb, PVE: 6.4%), and chr10 (*ca.* 6.8 Mb, PVE: 4.5%); however, their responsible genes are yet to be identified. Zhao *et al.* (2016) performed GWAS for grain and biomass-related plant architecture traits in sorghum using 315 genotypes comprising a subset of the SAP and found that significant SNPs on chr6 associated with GNP were located within a gibberellin (GA) biosynthetic gene similar to *Ent-kaurene synthase* (KS3), explaining 9.1% of the variation. Nevertheless, no natural variants or mutants that increase GNP as much as the *msd* mutants described above have been reported at this time.

More than 100 QTLs have been reported for TGW (Mace *et al.* 2019). Zou *et al.* (2020) recently identified *Sobic.001G341700* as the gene responsible for *qTGW1a*; notably, this gene is the same as *GC1*, which is the ortholog of rice *GS3*. Although rice *GS3* is a major gene for grain size that can explain 35–79% of the phenotypic variations in 180 rice varieties (Fan *et al.* 2006, 2009), the phenotypic variations in grain size contributed by sorghum *GC1*/*qTGW1a* are only 4–10% (Zou *et al.* 2020), suggesting that *GC1*/*qTGW1a* is a minor gene for variations in sorghum grain weight. The other QTL for sorghum grain weight, *qGW1*, was ﬁne mapped into a 101 kb on the short arm of chr1 using an F_2_ population derived from a cross between the grain sorghum variety SA2313 and the Sudan-grass variety Hiro-1 (Han *et al.* 2015); *qGW1* could explain 20–40% of phenotypic variations across multiple genetic backgrounds and environments. A gene (*Sobic.001G038900*), encoding a DUF567 protein, with high expression at the anthesis stage, was identified as the candidate gene responsible for *qGW1*. From GWAS using 390 diverse accessions comprising a subset of the SAP, Boyles *et al.* (2016) reported that 36, 53, and 19 SNPs were significantly associated with GY, GNP, and TGW, respectively. Notably, there were no overlapping loci for GNP and TGW, indicating that these traits may be independently improved to increase grain yield.

## Traits related to biomass yield

**Fig. 2.**
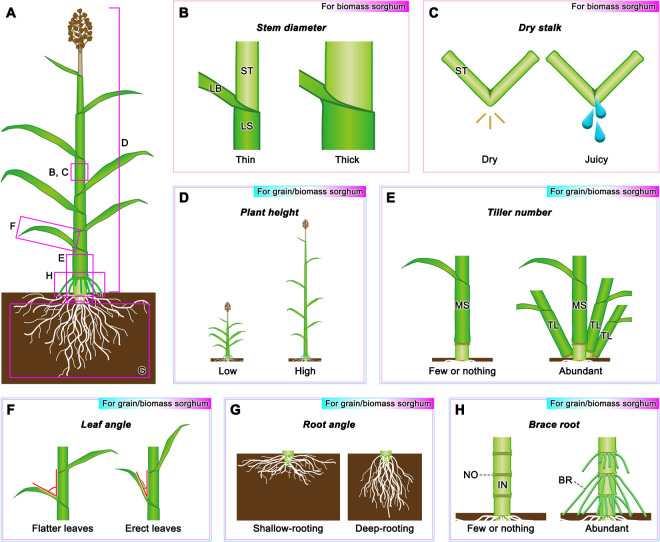
Useful traits for biomass sorghum and multiple end-uses. (A) Scheme of the sorghum plant. (B, C) Examples of useful traits for biomass sorghum: stem diameter (B) and dry stalk (C). LB: leaf blade, LS: leaf sheath, and ST: stem. (D–H) Useful traits for both grain and biomass sorghum: plant height (D), tiller number (E), leaf angle (F), root angle (G), and brace root (H). MS: main stem, TL: tiller, NO: node, IN: internode, and BR: brace root.

Sorghum biomass has a variety of uses such as forage, bioenergy feedstock for biofuels, bioproducts, and building materials. For these applications, a higher biomass yield per unit area is preferable. Although regulation of components such as lignin is also important when biomass is used for forage or material production, this section will not discuss the control of these components (see review in Li *et al.* 2008), but only the morphogenetic traits related to sorghum biomass (Fig. 2). Plant height and tiller number will be presented in a later section. Flowering time (duration of vegetative growth) also significantly affected biomass (see review in Mullet 2017), but this will not be discussed here.

### Stem diameter

Stem diameter is important not only because it contributes to the increase in biomass but also because it is related to the strength of the plant body and lodging resistance (Fig. 2B). Eight reproducible QTLs for stem diameter (ranging from 1.33 to 2.66 cm) have been reported on chr2 (*ca.* 59.1 Mb, PVE: 9.5%), chr3 (*ca.* 33.9 Mb, PVE: 13.3% and *ca.* 65.9 Mb, PVE: 7.6%), chr4 (*ca.* 51.4 Mb, PVE: 6.8%), chr6 (*ca.* 50.0 Mb, PVE: 10.2%), chr7 (*ca.* 7.9 Mb, PVE: 9.1%), chr8 (*ca.* 56.6 Mb, PVE: 7.5%), and chr9 (*ca.* 58.7 Mb, PVE: 8.1%) using a RIL population derived from a cross between sorghum varieties SS79 and M71 (Shiringani *et al.* 2010). Kong *et al.* (2020) performed QTL analysis for stem diameter in three portions, basal (ranging from 3.05 to 29.40 mm), middle (ranging from 1.18 to 25.20 mm), and rachis (ranging from 0.88 to 19.36 mm) using a sorghum RIL population derived from a cross between BTx623 and IS3620c; and reported six (chr1 *ca.* 22.3 Mb, PVE: 3.0%; chr3 *ca.* 61.9 Mb, PVE: 4.1%; chr6 *ca.* 46.1 Mb, PVE: 5.2%; chr6 *ca.* 59.3 Mb, PVE: 7.2%; chr7 *ca.* 59.5 Mb, PVE: 3.8% and chr8 *ca.* 52.3 Mb, PVE: 5.9%), six (chr1 *ca.* 53.0 Mb, PVE: 3.4%; chr1 *ca.* 58.2 Mb, PVE: 4.7%; chr6 *ca.* 50.4 Mb, PVE: 2.4%; chr6 *ca.* 56.6 Mb, PVE: 2.3%; chr7 *ca.* 58.4 Mb, PVE: 8.1% and chr8 *ca.* 52.3 Mb, PVE: 5.0%), and five (chr1 *ca.* 7.5 Mb, PVE: 4.0%; chr1 *ca.* 68.2 Mb, PVE: 7.0%; chr6 *ca.* 1.5 Mb, PVE: 3.2%; chr6 *ca.* 54.4 Mb, PVE: 3.8% and chr8 *ca.* 52.4 Mb, PVE: 3.1%) QTLs, respectively. Wang *et al.* (2022a) identified two genes (*Sobic.003G047700* and *Sobic.003G047800*), both encoding cytokinin-O-glucosyltransferase 3, and *Sobic.003G375100* encoding a mitochondrial DNA repair RAD52-like protein 1, as candidate genes responsible for stem diameter regulation using GWAS and RNA-seq. Since multiple mutants with thicker stems have been found in a gamma-ray-induced mutant library (Ordonio *et al.* 2014, 2016), it is expected that the identification of the genes responsible for these mutants will provide useful information for breeding stem thickness in the future.

### Dry stalk

Sweet sorghum varieties produce sugar juice in their stems, which can be used for sugar and ethanol production. Sorghum varieties can be classified into two groups according to the water content in the stem: juicy-stem and dry-stem varieties (Teshome *et al.* 1997, Fig. 2C). For over 100 years, this trait has been known to be regulated by a single locus, referred to as *D* (Hilson 1916, Swanson and Parker 1931). The responsible gene for *D* was recently cloned as *D*/*Dry* (*Sobic.006G147400*) and revealed that it encodes a NAC transcription factor which is the master switch inducing programmed cell death in the stem pith parenchyma (Casto *et al.* 2018, Fujimoto *et al.* 2018, Xia *et al.* 2018, Zhang *et al.* 2018). Although the juicy variety is suitable for bioethanol production, it has disadvantages lower lodging resistance and stem strength (Xia *et al.* 2018); therefore, these traits should be used appropriately according to the purpose.

## Traits related to various end-uses

The following traits are important for both biomass and grain sorghum varieties. Abiotic and biotic stress tolerance or resistance are also important traits that cannot be avoided while considering practical applications (see review in Anami *et al.* 2015) but will not be discussed here.

### Plant height

When considering plant height phenotypes, each favorable trait is used in the breeding process. Semi-dwarf phenotypes are valuable for grain production because they reduce the risk of lodging and increase the efficiency of mechanical harvesting. In contrast, non-dwarf phenotypes are essential for increasing biomass yields (Fig. 2D). Four major plant height-controlling loci (*Dw1–Dw4*) were identified in 1954 and have been reported that their recessive alleles reduce internode length (Quinby and Karper 1954); these have mainly been used in breeding for plant height in sorghum. The responsible genes for three out of the four loci have been identified (*Dw1*: *Sobic.009G230800*, *Dw2*: *Sobic.006G067700*, *Dw3*: *Sobic.007G163800*), which have advanced our knowledge of the molecular mechanisms involved in plant height regulation in sorghum. *Dw1* regulates the length of internodes by controlling cell proliferation and encodes a positive regulator gene of brassinosteroid (BR) signaling (Hilley *et al.* 2016, Hirano *et al.* 2017, Yamaguchi *et al.* 2016). *Dw2* encodes a protein kinase that is homologous to the AGCVIII protein kinase KIPK (Hilley *et al.* 2017), and *Dw3* encodes an ABCB1 auxin efflux transporter (Multani *et al.* 2003). Although progress has been made, the responsible gene for *Dw4* is yet to be identified. Recently, Hashimoto *et al.* (2021) reported a new plant height-related gene, *Dw7a* (*Sobic.007G137101*), encoding an R2R3 type MYB transcription factor. Similar to other *Dw* genes, the recessive alleles of *Dw7a* also reduced the internode length. It is interesting to note that the GA-related genes which facilitated semi-dwarf breeding in rice and wheat (responsible for the green revolution) are absent among the major dwarfing genes used for sorghum breeding. One of the reasons could be the loss-of-function mutations in genes involved in the early steps of GA biosynthesis cause abnormal and unsuitable culm bending in sorghum (Ordonio *et al.* 2014).

Although many other plant height-related loci have been detected by QTL analysis and GWAS in sorghum (Mace *et al.* 2019), most of the responsible genes for these loci have not been identified. Further research should focus on identifying these genes and utilizing such information in breeding programs.

### Tiller number

Tillering can contribute to the biomass and grain yield of sorghum (Lafarge *et al.* 2002, Fig. 2E). However, the physiological basis of tillering and its regulation are not fully understood as tillering is a complex trait strongly affected by both genotype and environment (Kim *et al.* 2010). The sorghum ortholog of the maize domestication gene *tb1* (*Sobic.001G121600*) is also involved in the regulation of sorghum tiller number (Kebrom *et al.* 2006). Jin *et al.* (2021) reported QTLs for tiller number (ranging from 0.0 to 4.0) located on chr1 (*ca.* 57.0 Mb, PVE: 10.2% in 2019), chr3 (*ca.* 9.4 Mb, PVE: 10.8% in 2019), chr5 (*ca.* 61.4 Mb, PVE: 13.8% in 2019), chr6 (*ca.* 50.0 Mb, PVE: 25.0% in 2019), chr7 (*ca.* 56.7 Mb, PVE: 13.5% in 2019), chr8 (*ca.* 45.1 Mb, PVE: 44.3% in 2018), and chr9 (*ca.* 44.3 Mb, PVE: 38.7% in 2018 and *ca.* 51.8 Mb, PVE: 20.1% in 2019) using a RIL population derived from a cross between sorghum Tx623A and sudangrass Sa. Using GWAS, Luo *et al.* (2020) identified *Sobic.009G164600*, encoding a C2H2 zinc ﬁnger protein, as a potential candidate gene for tiller number regulation. Maize *tin1*, which also encodes a C2H2 zinc ﬁnger protein, has been shown to control tiller number (Zhang *et al.* 2019); *Sobic.009G164600* is likely involved in the control of tiller number in sorghum, although further studies are needed to confirm its role. Chen *et al.* (2018) identified and functionally characterized a mutant of the *Non-dormant Axillary Bud 1* (*NAB1*) gene in an EMS-mutagenized sorghum population. Map-based cloning revealed that *NAB1* (*Sobic.006G170300*) encodes a carotenoid cleavage dioxygenase that is thought to be involved in strigolactone biosynthesis; it is primarily expressed in axillary nodes and tiller bases to reduce tiller number (Chen *et al.* 2018). The *nab1* mutant, which shows increased tiller number and reduced plant height, may serve as a useful tool for future tiller number improvement. In addition, the fact that multiple high-tillering mutants have also been found in the gamma-ray-induced mutant library indicates that tiller number is a helpful trait that still has room for improvement (Ordonio *et al.* 2016).

### Leaf angle

Leaf angle, the inclination between the leaf blade and the stem, is one of the most important architectural traits in cereals, as it is directly related to biomass and grain yield (Ort *et al.* 2015, Zelitch 1982, Fig. 2F). Most mutant studies involving leaf angle in model species have shown that leaf angle is altered by allelic changes in BR biosynthesis and/or signaling genes (Tanaka *et al.* 2009, Wada *et al.* 1981, Wang *et al.* 2008, Yamamuro *et al.* 2000). In addition to BR, certain studies have indicated the involvement of other phytohormones such as auxin, ethylene, abscisic acid, and GA in leaf angle determination (Cao and Chen 1995, Shimada *et al.* 2006, Xu *et al.* 2014). BR-related genes have also been associated with natural variations in the leaf angle in sorghum (Perez *et al.* 2014). *Dw3*, a well-known auxin transporter gene that has a major effect on sorghum plant height, also has pleiotropic effects on leaf angle (Truong *et al.* 2015). Some studies have detected similar loci associated with *Dw3* involved in the regulation of leaf angle (Mantilla-Perez *et al.* 2020, Natukunda *et al.* 2022, Zhi *et al.* 2022); however, QTL analysis using a RIL population in which both parents carried the same *Dw3* allele also detected a QTL for leaf angle in this region (Truong *et al.* 2015), suggesting that another gene close to *Dw3* may also be involved in leaf angle regulation in sorghum. RNA-seq data suggested that *Sobic.007G165800* (BAHD acyltransferase DCR, a homolog of rice *Slender Grain*), *Sobic.007G166900* (*WALLS ARE THIN 1*), *Sobic.007G175600* (small auxin up‑regulated RNA 36), *Sobic.007G153001* (potassium ion transporter), and *Sobic.007G160400* (zinc‑finger homeodomain protein 2) are novel candidate genes co-localized with QTL in the *Dw3* region (Natukunda *et al.* 2022). Combining the results of GWAS and QTL analysis with RNA-seq data, the following eleven candidate genes responsible for leaf angle regulation were identified: *Sobic.001G161500* (Aux/IAA transcription factor IAA12), *Sobic.001G166401* (homolog of rice GA20 oxidase 4, which catalyzes oxidation steps during GA biosynthesis), *Sobic.001G170301* (homologous to the GA-stimulated transcript *OsGASR2*), *Sobic.001G172400* (cytochrome P450 85A1 homologous to the rice *brassinosteroid-deficient dwarf1* that catalyzes the C6 oxidation step during BR biosynthesis), *Sobic.002G353200* (BZR1/BES1 transcription factor), *Sobic.003G036700* (homolog of *OsCKX1*, which encodes a cytokinin dehydrogenase 1), *Sobic.003G096100* (*WALLS ARE THIN 1*), *Sobic.003G133800* (leucine-rich repeat protein associated with BRASSINOSTEROID INSENSITIVE 1-ASSOCIATED RECEPTOR KINASE 1), *Sobic.004G178000* (homologous to *OsRR2*, the predicted negative regulator of cytokinin signaling), *Sobic.005G030400* (cytochrome P450 90A1, homologous to *OsCPD1*/*2*), and *Sobic.006G091700* (putative 12-oxophytodienoate reductase involved in JA biosynthesis) (Mantilla-Perez *et al.* 2020, Natukunda *et al.* 2022). The sorghum leaf arrangement is currently far from that proposed in the “smart canopy” model (Mantilla-Perez *et al.* 2020). Further studies are needed to understand the regulatory mechanisms involved in leaf angle to improve the sorghum leaf arrangement.

### Root angle

Deep rooting, associated with crown (nodal) root angle, is thought to help plants avoid drought stress by extracting water from deeper layers of soil (Fukai and Cooper 1995, Gowda *et al.* 2011, Uga *et al.* 2013, Fig. 2G). To date, only a few genetic studies on root angle have been reported in sorghum. Mace *et al.* (2012) reported four QTLs for nodal root angle (ranging from 14.6 to 32.2°) in sorghum on chr5 (*ca.* 36.1 Mb, PVE: 10.0% and *ca.* 65.0 Mb, PVE: 29.8%), chr8 (*ca.* 30.5 Mb, PVE: 6.7%), and chr10 (*ca.* 58.8 Mb, PVE: 11.7%) using a RIL population derived from a cross between B923296 and SC170-6-8. All four root angle QTLs co-located with previously identified QTLs for stay-green (a drought-adaptive trait characterized by a distinct green leaf phenotype during grain filling under drought conditions), indicating a strong relationship between root angle and stay-green (Borrell *et al.* 2014, Mace *et al.* 2012). In the case of gravitropism response, which is closely related to the root angle, the asymmetric accumulation of auxin suppresses cell elongation on the lower side of the root (Abas *et al.* 2006). Therefore, it is plausible to consider auxin-related genes as candidates for the control of root angle. Lopez *et al.* (2017) detected a root angle QTL on sorghum chr3 (*ca.* 4.9 Mb, PVE: 2.6%) using a biparental mapping population generated from an initial cross of the parents Bk7 and Early Hegari-Sart. They suggested that *Sobic.003G052700*, encoding a putative tryptophan aminotransferase, was a strong candidate gene for root angle trait due to its involvement in the indole-3-pyruvic acid auxin biosynthesis pathway (Lopez *et al.* 2017). Since deep rooting and/or root angle are important traits for sorghum, which is also expected to grow in drought-prone areas, further research is required to study these aspects in the future.

### Brace root

Brace roots, often found in maize and sorghum, are a type of nodal root initiating from the aerial stem nodes (Fig. 2H). Studies have demonstrated that brace roots contribute enormously to lodging resistance through effective anchorage and water/nutrient uptake during late growth in maize (Varney and Canny 1993, Wang *et al.* 1994). Numerous genetic studies have been conducted on brace roots in maize, and several genes that control this trait have been identified. For example, loss-of-function of *ROOTLESS CONCERNING CROWN AND SEMINAL ROOTS* (*RTCS*, *GRMZM2G092542*), which encodes a LOB domain protein, causes defects in seminal, crown, and brace root primordia formation; thus, the maize *rtcs* mutant is devoid of brace roots (Hetz *et al.* 1996, Taramino *et al.* 2007). A knockdown mutant of *TEOSINTE GLUME ARCHITECTURE 1* (*TGA1*), which encodes a squamosa-promoter binding protein, showed increased number of nodes with brace roots (Wang *et al.* 2015), indicating that *TGA1* is also involved in brace root formation in maize. In the case of sorghum, only one study has performed QTL analysis for the presence of brace roots; Li *et al.* (2014) detected two QTLs on chr6 (*ca.* 22.7 Mb, PVE: 7.0%) and chr7 (*ca.* 57.8 Mb, PVE: 52.5%) using an F_2_ population derived from a cross between Sansui with brace roots and Jiliang 2 with very few brace roots. The QTL on chr7 had a significant effect, but the responsible gene has not been identified. Because brace roots are essential for both grain and biomass sorghum, it is necessary to identify the loci/genes that control brace root development using numerous analytical populations for future utilization of this trait.

## Crop ideotypes

For biomass sorghum, a single-culm variety with a plant height exceeding 6 m may have a great visual impact, but it may not be ideal because of the high risk of lodging. Given the risk of lodging, the ideal variety for biomass sorghum might be one with moderate plant height, thick stems with abundant tillering, erect leaves, and vigorous brace roots. The ideal variety for grain sorghum might be one with a lower plant height suitable for mechanical harvesting, moderate tillering without grain yield reduction, large grain size, fertile PSs, erect leaves, and vigorous brace roots for lodging resistance. However, it is not appropriate to suggest that “this variety is perfect” because the required traits such as the presence or absence of awns and size of the glume may vary depending upon the cultivation environment. In practice, there are trade-offs among traits; for example, it is known that tiller number is negatively correlated with stem diameter in sorghum germplasms (Wang *et al.* 2022a). In addition, many agronomic traits are affected by the environment, making it difficult to breed the desired variety as desired; therefore, varieties for either end use will eventually require fine-tuning based on the environment and cultivation requirements.

## Conclusions and future perspectives

It has to be said that although great progress has been made in sorghum genetic research in the past few decades, the accumulation of information on loci/genes controlling various traits in sorghum (identified genes are listed in Table 1) is still quite poor compared to those in rice and maize. By enhancing our knowledge of the multiple loci/genes controlling important traits, it will be possible to increase the number of breeding options, such as examining combinations of loci that minimize undesirable trade-offs between traits. Therefore, for full use of sorghum in the future, we should vigorously collect information on the loci/genes controlling each trait. Although GWAS is a powerful tool, it will be essential to continue traditional/biparental QTL analysis by picking up accessions that show characteristic phenotypes for traits of interest because biparental QTL mapping can sometimes be more efficient than GWAS in detecting rare alleles in populations. While it takes a lot of time and care to prepare mapping populations, analysis using multiparent populations such as MAGIC (Multi-parent Advanced Generation Intercrosses) and NAM may be highly powerful for traits regulated by complex networks (Huang *et al.* 2015, Yu *et al.* 2008). Therefore, these methods should be used more than ever in future sorghum research.

Rapid innovations in next-generation sequencing techniques, such as restriction site-associated DNA sequences (Baird *et al.* 2008), have made genome-wide high-density DNA marker information readily and inexpensively available, allowing for higher-resolution QTL analysis and GWAS to further advance sorghum gene hunting. Furthermore, whole-genome re-sequencing data for 400 sorghum accessions have recently been reported (Boatwright *et al.* 2022), and GWAS using this ultra-high-density marker information is expected to accelerate sorghum genomic research. In addition to the above re-sequencing data, with the availability of the sorghum gene expression atlas (Makita *et al.* 2015, Shakoor *et al.* 2014), the recent completion of sorghum pan-genomes (Tao *et al.* 2021), and the development of SorghumBase online (Gladman *et al.* 2022), we are entering an exciting new era of sorghum genomic research. Although there are issues such as low efficiency and time consumption that are yet to be resolved, transformation and genome editing are also possible in sorghum (see review in Silva *et al.* 2022). By utilizing these critical resources/technologies and accelerating breeding, sorghum will become a vital crop contributing to the achievement of SDGs.

## Author Contribution Statement

H.T. wrote the manuscript.

## Figures and Tables

**Table 1.** Identified responsible genes for morphological traits in sorghum

Trait	Gene	Gene ID (v3.1.1)	Annotation	References
Seed shattering	*Sh1*	*Sobic.001G152901*	YABBY transcription factor	Lin *et al.* (2012)
	*SbWRKY*	*Sobic.001G148000*	WRKY transcription factor	Tang *et al.* (2013)
Grain number	*MSD1*	*Sobic.007G135700*	TCP transcription factor	Jiao *et al.* (2018)
	*MSD2*	*Sobic.006G095600*	13-lipoxygenase	Gladman *et al.* (2019)
	*MSD3*	*Sobic.001G407600*	Linoleic desaturase FAD7	Dampanaboina *et al.* (2019)
	*KS3*	*Sobic.006G211400*	Ent-kaurene synthase	Zhao *et al.* (2016)
Grain weight	*qGW1*	*Sobic.001G038900*	Expressed protein (DUF567)	Han *et al.* (2015)
	*qTGW1a*	*Sobic.001G341700*	G protein γ subunit	Zou *et al.* (2020)
Glume coverage	*GC1*	*Sobic.001G341700*	G protein γ subunit	Xie *et al.* (2022)
Awn presence	*awn1/DAI*	*Sobic.003G421300*	ALOG transcription factor	Zhou *et al.* (2021), Takanashi *et al.* (2022)
Dry stalk	*D/Dry*	*Sobic.006G147400*	NAC transcription factor	Xia *et al.* (2018), Fujimoto *et al.* (2018), Zhang *et al.* (2018), Casto *et al.* (2018)
Plant height	*Dw1*	*Sobic.009G230800*	Positive regulator of BR signaling	Hilley *et al.* (2016), Yamaguchi *et al.* (2016)
	*Dw2*	*Sobic.006G067700*	AGCVIII protein kinase KIPK	Hilley *et al.* (2017)
	*Dw3*	*Sobic.007G163800*	ABCB1 auxin efflux transporter	Multani *et al.* (2003)
	*Dw7a*	*Sobic.007G137101*	MYB transcription factor	Hashimoto *et al.* (2021)
Tiller number	*tb1*	*Sobic.001G121600*	TCP transcription factor	Kebrom *et al.* (2006)
	*NAB1*	*Sobic.006G170300*	Carotenoid-cleavage dioxygenase 7	Chen *et al.* (2018)
Leaf angle	*Dw3*	*Sobic.007G163800*	ABCB1 auxin efflux transporter	Truong *et al.* (2015)
